# Personality traits and hardiness as risk- and protective factors for mental distress during the COVID-19 pandemic: a Norwegian two-wave study

**DOI:** 10.1186/s12888-022-04237-y

**Published:** 2022-09-15

**Authors:** Espen Rasmussen Lassen, Kristen Hagen, Gerd Kvale, Jarle Eid, Stephanie Le Hellard, Stian Solem

**Affiliations:** 1 Department of Psychology, Norwegian University of Science and Technology, Trondheim, Norway; 2 Molde Hospital, Department of Psychiatry, Møre og Romsdal Hospital Trust, Molde, Norway; 3 Bergen Center for Brain Plasticity, Haukeland University Hospital, Bergen, Norway; 4 Department of Mental Health, Norwegian University of Science and Technology, Trondheim, Norway; 5 Department of Clinical Psychology, University of Bergen, Bergen, Norway; 6 Department of Psychosocial Science, University of Bergen, Bergen, Norway; 7 Center for Crisis Psychology, University of Bergen, Bergen, Norway; 8 NORMENT, Department of Clinical Science, University of Bergen, Bergen, Norway; 9 Department of Medical Genetics, Haukeland University Hospital, Bergen, Norway

**Keywords:** Personality traits, Hardiness, Anxiety, Depression, COVID-19 pandemic

## Abstract

**Background:**

Several risk factors for anxious-depressive symptomatology during the COVID-19 pandemic have been established. However, few studies have examined the relationship between personality traits, *hardiness*, and such symptomatology during the pandemic. These constructs might serve as risk- and/or protective factors for such mental distress through the pandemic.

**Methods:**

A sample of 5783 Norwegians responded to a survey at two time points within the first year of the pandemic. The first data collection was in April 2020 (T1) and the second in December 2020 (T2). Measures included the Ten-Item Personality-Inventory, the Revised Norwegian Dispositional Resilience Scale, and the Patient Health Questionnaire Anxiety and Depression Scale. Analyses were performed using Pearson’s correlations, multiple linear regression, and a moderation analysis.

**Results:**

Anxious-depressive symptomatology in early phases (T1) of the pandemic was the strongest predictor for the presence of such symptomatology 9 months after the outbreak (T2). Personality and *hardiness* correlated significantly with mental distress at T1 and T2. Personality traits explained 5% variance in symptoms when controlling for age, gender, solitary living, negative economic impact, and mental distress at baseline. Higher *neuroticism* predicted higher mental distress, whereas higher *conscientiousness* and *extraversion* predicted less mental distress. *Hardiness* did not explain variance in outcome beyond personality traits. *Hardiness* did not significantly moderate the relationship between *neuroticism* and mental distress.

**Conclusion:**

Individuals with high levels of neuroticism had greater difficulties adapting to the circumstances of the COVID-19 pandemic and were more prone to mental distress. Contrastingly, higher *conscientiousness* and *extraversion* may have served as protective factors for mental distress during the pandemic. The current findings might aid identification of vulnerable individuals and groups. Consequently, preventive interventions could be offered to those who need it the most.

**Supplementary Information:**

The online version contains supplementary material available at 10.1186/s12888-022-04237-y.

## Background

Several meta-analyses and systematic reviews have reported elevated rates of anxious-depressive symptomatology during the COVID-19 pandemic [1–5]. For instance, the prevalence of anxiety and depression reported by Luo and colleges [2] was 33 and 28%, respectively, while Cénat and colleges [1] found lower rates (15 and 16%, respectively). Prevalence estimates of self-reported anxiety and depression before the pandemic was 14.7% [6] and 8.1% [7], respectively. Although there are temporal and cultural differences in the referred studies, the pandemic and its consequences could appear to impact mental health. However, despite ascending prevalence of anxious-depressive symptomatology in early phases of the pandemic, rates of suicide, life satisfaction and loneliness have remained constant throughout the first year of the pandemic [8]. This suggests a complex picture of stressors and adaptive responses. Consequently, more knowledge concerning risk- and protective factors for anxious-depressive symptomatology during the pandemic is warranted.

Numerous risk factors for symptoms of anxiety and depression during the pandemic have been identified, including solitary living, lower age, pre-existing mental health problems, social isolation/quarantine, low socioeconomic status, negative economic influence, poor sleep quality, and female gender [2, 4, 5, 8, 9]. Although the Big Five personality traits [10] are involved in the etiology of anxiety and depression [11–13], research on personality as a risk- and protective factor for such symptoms during the COVID-19 pandemic is scarce. Preceding the pandemic, the relationships between higher *neuroticism*, lower *conscientiousness*, and higher anxiety and depression were already well established [11–13]. Lower *extraversion* was also implicated [13], although less consistently [12]. During the COVID-19 pandemic, one might expect lower *neuroticism* and higher *conscientiousness* to predict less anxious-depressive symptomatology through an inclination towards emotional stability, positive emotions, gregariousness, and self-competence [14]. The role of *extraversion* could be more questionable during the pandemic, as in-person social contact has been restricted.

As far as we are aware, only a handful of studies regarding personality, anxiety and depression have been conducted during the COVID-19 pandemic. Summarized, cross-sectional results have established a positive relationship between higher *neuroticism* and higher levels of psychological distress [15–20]. Furthermore, *extraversion*, *agreeableness*, *openness,* and *conscientiousness* are cross-sectionally associated with favorable psychological adjustment, and less anxious-depressive symptoms [15, 18]. Noticeably, *neuroticism* predicted more worry and risk-perception of the pandemic [17]. However, more large-scale studies exploring if and how personality traits are associated with anxious-depressive symptomatology during the COVID-19 pandemic are warranted.

As emphasized by Klein et al. [11] it is necessary to develop explanatory models of how and why personality and psychopathology are interrelated. The construct of resilience might be a central interactional mechanism between personality and psychopathology, as the protective model of resilience [21] postulates that resilience moderates the relationship between risk factors (e.g., *neuroticism*) and adverse outcomes (e.g., anxious-depressive symptomatology). Furthermore, a recent meta-analysis has established relationships between the Big Five personality traits and resilience [22]. Results revealed that resilience was negatively related to *neuroticism* and positively related to *extraversion*, *openness*, *agreeableness*, and *conscientiousness*. Moreover, studies have established positive relationships between resilience factors and well-adjusted personality profiles [23], in addition to personality clusters that corresponds to resilient self-regulation [24]. Withal, *neuroticism* is related to heightened stress [25, 26], and resilience is known to moderate negative outcomes of stress [21, 27]. Hence, there might be interactional effects of personality traits and resilience on mental distress.

There are several ways in which resilience can be defined and conceptualized [28–30]. *Hardiness* [31, 32] is a conceptualization of trait resilience, pertaining to “a generalized style of functioning characterized by a strong sense of *commitment*, *control*, and *challenge* that serves to mitigate the negative effects of stress” [33 , p. 237]. Kobasa [31] described *commitment* as an inclination towards active involvement in one’s activities, whereas *control* involves the belief in one’s ability to influence important life events. Moreover, *challenge* concerns a disposition towards considering changing circumstances as natural and as a chance for development and growth. *Commitment* is contrasted with alienation, *control* with powerlessness, and *challenge* with threat [34].

Measured with the Dispositional Resilience scale-15 (DRS-15) [35], studies have demonstrated that *hardiness* buffers against depressive symptoms [36], post-traumatic stress symptoms [37], and somatic and psychological symptoms [38]. Meta-analytically, *hardiness* is positively associated with *extraversion*, active coping, social support, self-efficacy [39], and negatively related to negative affectivity*, neuroticism,* and trait anxiety [40]. Hence, resilient individuals might cope with adverse life events by perceiving stressors as less threatening, assuming control over important outcomes in one’s life, utilizing adaptive coping strategies and social support [32, 40, 41]. Consequently, this might reduce such individuals’ risk of experiencing mental distress during adverse life events throughout the COVID-19 pandemic.

Research on *hardiness* during the pandemic is scarce. A cross-sectional investigation of 510 Italian emergency workers found that *hardiness* buffers against the effects of stress on secondary trauma [42]. However, this study did not include measures of both personality traits and *hardiness*, in relation to mental distress. To the best of our knowledge, no such studies have yet been issued in the context of the COVID-19 pandemic.

Thus, the aim of the current study is to examine *hardiness*, personality traits, and their relationship to mental distress in a heterogeneous Norwegian sample, during two phases of the COVID-19 pandemic. More specifically, the study had two aims. First, to study the predictive validity of *hardiness* and personality traits for mental distress. Second, to study whether *hardiness* moderated the hypothesized relationship between *neuroticism* and mental distress. First, we hypothesized that *neuroticism* would predict more mental distress. Second, *hardiness* was hypothesized to predict lower levels of mental distress. Third, based on the established relationships between the Big Five personality traits and resilience, as well as the protective resilience-model [21], *hardiness* was hypothesized to moderate the relationship between *neuroticism* and anxious-depressive symptomatology. We expected *hardiness* to reduce the hypothesized positive relationship between *neuroticism* and such symptoms.

## Method

### Participants and procedure

This study is a part of the BryDeg2020-project [43], which is a longitudinal survey study examining mental health in the Norwegian population during the COVID-19 pandemic. The study obtained ethical approval from the Regional Committee for Medical and Health Research Ethics in Northern Norway (REK Nord, reference number 123324), and was launched shortly after the societal lockdown in Norway (12th of March 2020). Data was collected in two waves, using online surveys. Wave 1 (T1) lasted from 1st of April to 2nd of June 2020 (Most data collected in April [87.2%]). Wave 2 (T2) was carried out between December 1st and 22nd 2020. Recruitment to T1 was accomplished as several organizations contributed by sharing the survey invitation with their members. This included most Universities and Colleges in Norway, the national student association, the Norwegian Council for Mental Health, and one of Norway’s largest hospital trusts. Furthermore, the study was advertised in social media, and the Norwegian Broadcasting Corporation encouraged the public to participate. At T1, participants were asked to take part in the T2-data collection. Follow-up questionnaires were sent to those who agreed (*n =* 13,410, 69% of the original sample).

Informed consent was obtained from all participants before inclusion. At T1 the sample consisted of 19,372 participants, of whom 6017 (31.06% of the sample at T1) responded to the follow-up at T2. The study has an overrepresentation of women and young adults with high levels of education compared with the general Norwegian population [9]. Mental distress was marginally higher in subjects participating in T1 only, compared to those who participated in both waves and were included in this study (*t* = 4.95, *p <* .001, *d =* 0.08; see next paragraph). The groups’ hardiness-scores did not differ (*t =* − 1.44, *p =* 0.15, *d =* − 0.02). Furthermore, as noted by Unnarsdóttir et al. [44], the dropouts were characterized by lower age, student status, fewer with a completed bachelor’s degree, and there were more men.

The analyses in the current study are based on data from subjects who participated at both at T1 and T2. Of the participants who responded at both waves, 193 (3.21%) had missing data on one or more of the utilized measures. Overall, there were 266 (0.74%) values missing. As missing rates below 5% are considered inconsequential [45], participants with missing data were trimmed list-wise. Consequently, 106, 41, and 46 cases were deleted from the *hardiness* inventory, the depression scale, and the anxiety measure, respectively. Participants with an unreliable response style (*n =* 41) were also trimmed list-wise. Accordingly, the final sample consisted of 5783 participants. Table 1 displays sample demographics at both times of measurement.

**Table 1:** Sample demographics and descriptive statistics of included measures (*N* = 5783)

	T1		T2	
Variable	*%*	*n*	*%*	*n*
Gender				
Male	21.39	1237	21.39	1237
Female	78.07	4515	78.07	4515
Other	0.54	31	0.54	31
Student	51.51	2979	–	–
Self-reported psychiatric disorder	31.90	1845	–	–
Increased alcohol use	18.43	1066	–	–
Less exercise	35.86	2074	–	–
Lost job/leave	2.47	143	–	–
Bachelor’s degree +	53.80	3111	–	–
Neg. economic impact	21.55	1246	24.99	1445
Solitary living	22.00	1272	22.67	1311
	*M*	*SD*	*M*	*SD*
Age	34.35	13.43	34.88	13.39
PHQ-ADS	14.25	10.94	15.13	10.77
DRS-15-R	27.91	6.72	–	–
DRS-15-R Commitment	9.58	3.40	–	–
DRS-15-R Control	10.57	2.62	–	–
DRS-15-R Challenge	7.76	3.09	–	–
Neuroticism	–	–	3.42	1.52
Conscientiousness	–	–	5.49	1.23
Extraversion	–	–	4.47	1.54
Agreeableness	–	–	5.07	1.06
Openness	–	–	5.02	1.14

*Note*. *DRS-15-R* = Revised Norwegian Dispositional Resilience Scale, *PHQ-ADS = * Patient Health Questionnaire Anxiety and Depression Scale; Bachelor’s degree + = highest finished education level equals bachelor’s degree or above. Personality traits were assessed with the Ten-Item Personality Inventory. All variables were measured using self-report instruments

### Measures

Personality traits were measured at T2 only, utilizing the Ten-Item Personality-Inventory (TIPI) [46]. The TIPI assesses the personality traits in Costa & McCrae’s Five-Factor Model [10], using two items per trait. Each item is scored on a 7-point scale (ranging from 1 = “strongly disagree” to 7 = “strongly agree”). The TIPI has demonstrated poor to good test-retest reliability (.52 to .83) dependent on the trait measured, and self-other agreement estimates ranges from .42 to .61 [46, 47], The instrument has adequate structure validity, convergent validity (.36 to .61 with the Revised NEO Personality Inventory) [14], and predictive validity [46, 47]. Reported Cronbach’s α coefficients ranges from .50 to .57 on average across traits [46–48]. In the current study, Cronbach’s α coefficients were .79 for *extraversion*, .69 for *conscientiousness*, .72 for *neuroticism*, .45 for *openness*, and .35 for *agreeableness*. The TIPI measures *emotional stability* (the opposite pole of *neuroticism*). However, to aid interpretability and comparison of results with similar studies, the *emotional stability*-variable was reversed to represent *neuroticism*.

*Hardiness* was measured with the Revised Norwegian Dispositional Resilience Scale (DRS-15-R) [33] at T1 only. The DRS-15-R is a valid and reliable measure of personal characteristics indicative of efficacious psychological adaption in the face of adverse life events [33]. The 15-item self-report questionnaire assesses three facets of *hardiness* (*commitment*, *control*, and *challenge*) on a Likert scale ranging from 0 (“not true”) to 3 (“completely true”). The DRS-15-R was measured at T1. In the current study, the overall Cronbach’s α value was .82, whereas α values were .83, .74 and .72 for *commitment*, *control*, and *challenge*, respectively.

As there is evidence for related underlying psychological constructs of anxiety and depression, as well as high comorbidity between the two disorder categories [49], the Patient Health Questionnaire Anxiety and Depression Scale (PHQ-ADS) [49] was used to assess mental distress at both times of measurement. The PHQ-ADS is a joint measure for symptoms of anxiety and depression, which is computed by summing the scores of the Patient Health Questionnaire-9 (PHQ-9) [50] and the Generalized Anxiety Disorder 7 (GAD-7) [51]. The scores of the PHQ-ADS range from 0 to 48, with greater scores indicating more severe symptoms. Scores of 0–9, 10–19, 20–29, and 30–48 are indicative of minimal, mild, moderate, and severe anxious-depressive symptomatology, respectively [49]. The PHQ-ADS is a reliable and valid measure with satisfactory psychometric properties [49, 52]. In the current study, Cronbach’s α values were .94 at both T1 and T2.

Housing situation was reported on a 5-point scale (1 = solitary living, 2 = with one or both parents, 3 = with roommates, 4 = with partner/wife/husband, 5 = other). Economic impact of the pandemic was similarly reported (1 = very negative, 2 = somewhat negative, 3 = no change, 4 = somewhat positive, 5 = very positive). Alcohol consumption and exercise were measured as two separate items, utilizing 5-point scales (from 1 = significantly reduced, to 5 = significantly increased). These measures were dichotomized, specifying whether subjects were living alone (score = 1) or not (score = 2–5), had increased alcohol consumption (score = 4 and 5) or not (score = 1–3), exercised less (score = 1 and 2) or not (score = 3–5), and experienced negative economic impact (score = 1 and 2) or not (score = 3–5). Participants reported changes in job status (1 = yes [on leave/lost job], 2 = no changes). Self-reported psychiatric disorder (and treatment) was measured as 0 = not received treatment, 1 = previously psychiatric treatment, and 2 = currently in treatment for psychiatric treatment. Participants that had received treatment were then asked to specify for which disorder. The reported variable grouped together scores 1 and 2, as to represent individuals who currently, or previously, received treatment for a psychiatric disorder.

### Statistical analyses

Pearson’s correlations were utilized to provide correlations of all study variables. Furthermore, hierarchical regression was applied to investigate hypotheses 1 and 2. The model controlled for age and gender on step 1, self-reported solitary living on step 2, self-reported negative economic impact on step 3, and symptoms at baseline on step 4. Personality traits were entered at step 5. *Hardiness* was entered at step 6. To test hypothesis 3, we administered an additive multiple moderation analysis, based on the least squares’ method. The model utilized the control variables from the regression analysis as covariates. To aid interpretability and prevent multicollinearity, all independent variables were mean centered before analyzed with inference statistics [53]. Dummy coding of the three-leveled gender variable was applied before entering the variable into the regression- and moderation analyses. The variable “female” was coded as 0 = non-female (i.e., male and other gender) and 1 = female. The variable “other gender” was coded as 0 = non-other (i.e., male and female gender) and 1 = other gender. To ensure that the results were uninfluenced by the trimming procedures, the correlation and regression analyses were repeated using multiple imputation. The imputation was performed using the multiple imputation algorithm with 5 simulations in IBM SPSS statistics version 26.

## Results

Table 1 displays descriptive statistics of the utilized measures. Levels of mental distress (PHQ-ADS) increased marginally from T1 to T2 (*t =* − 8.55, *p <* .001, *d =* − 0.08). Drawing on Kroenke and colleagues’ [49] cut-off scores, the prevalence of minimal, mild, moderate, and severe levels of anxious-depressive symptomatology at T1 was 41.74%, 30.69%, 15.91% and 11.65%, respectively. At T2, the prevalence estimate of anxious-depressive symptomatology was 37.45% (minimal), 31.92% (mild), 18.59% (moderate), and 12.04% (severe).

### Associations between hardiness, personality and symptoms of anxiety and depression

Table 2 is a correlation matrix with the present study’s variables. *Hardiness* and its subscales (measured at T1) were significant and negatively correlated with mental distress at both waves, as were *extraversion*, *agreeableness*, *conscientiousness*, and *openness* (measured at T2). There was a significant and positive relationship between *neuroticism* and mental distress at both waves. Results were essentially identical, with no differences in significance levels and merely negligible differences in a few correlation coefficients, when the correlation analysis was based on data imputed with multiple imputation (*N =* 5969; see supplemental Table 1).

**Table 2:** Correlations between hardiness, personality traits, and mental distress (*N* = 5783)

Variable	1	2	3	4	5	6	7	8	9	10
1. Commitment T1	–									
2. Control T1	.40*	–								
3. Challenge T1	.35*	.17*	–							
4. DRS-15-R total T1	.82*	.67*	.70*	–						
5. Neuroticism T2	−.39*	−.22*	−.35*	−.44*	–					
6. Extraversion T2	.36*	.16*	.32*	.39*	−.21*	–				
7. Agreeableness T2	.18*	.09*	.13*	.19*	−.21*	.13*	–			
8. Openness T2	.25*	.11*	.34*	.33*	−.15*	.31*	.18*	–		
9. Conscientiousness T2	.33*	.17*	.08*	.27*	−.26*	.17*	.21*	.08*	–	
10. PHQ-ADS T1	−.58*	−.27*	−.38*	−.57*	.53*	−.21*	−.12*	−.10*	−.27*	–
11. PHQ-ADS T2	−.49*	−.21*	−.30*	−.47*	.58*	−.24*	−.16*	−.10*	−.31*	.74*

*Note.* Variables 1–4 = revised Norwegian dispositional resilience scale; variables 5–9 = Ten-Item Personality-Inventory; *PHQ-ADS =* Patient Health Questionnaire Anxiety and Depression Scale, *T* *=* Wave number. Results were essentially identical, with no differences in significance levels and merely marginal differences in correlation coefficients, when the analysis was based on data using multiple imputation (*N =* 5969; see supplemental Table 1)

* *p <* .001

### Predicting anxious-depressive symptomatology using personality traits and hardiness

Table 3 presents the results of the multiple linear regression with PHQ-ADS at T2 as the outcome variable. Overall, the model explained 61% of the variance in symptomatology. Age and gender, solitary living, negative economic impact during the pandemic, symptoms at baseline, and personality traits all significantly explained variance in outcome. Personality traits uniquely explained 5% of the variance in symptoms, whereas *hardiness* did not significantly add to the explained variance. All individual variables were significant predictors of mental distress in the final step of the equation, except from female gender, other gender, *openness*, and *hardiness*. More specifically, greater age prospectively predicted less symptoms, whereas solitary living and negative economic impact both predicted more symptoms, cross-sectionally. Withal, higher anxious-depressive symptomatology at baseline was the strongest predictor of more symptoms at T2. Cross-sectionally, higher *neuroticism* predicted more mental distress, whereas higher *conscientiousness*, *agreeableness*, and *extraversion* predicted less mental distress.

**Table 3:** Predicting mental distress (T2) using personality traits and hardiness (*N* = 5783)

Step	*F*	*p*	*R*^*2*^_*adj.*_	*ΔR*^*2*^
1. Age & gender T1	227.85	<.001	.11	.11*
2. Solitary living T2	189.04	<.001	.12	.01*
3. Negative economic impact T2	214.00	<.001	.16	.04*
4. PHQ-ADS T1	1236.25	<.001	.56	.41*
5. TIPI T2	830.04	<.001	.61	.05*
6. DRS-15-R T1	761.05	<.001	.61	.00
Predictors in final step	*β*	*t*	*p*	
Age	−.06	−6.21	<.001	
Female gender	.02	1.82	.069	
Other gender	−.01	−.88	.381	
Solitary Living	.02	2.53	.011	
Negative economic impact	.07	8.45	<.001	
PHQ-ADS	.56	49.26	<.001	
Neuroticism	.23	21.85	<.001	
Conscientiousness	−.07	−8.27	<.001	
Agreeableness	−.02	−2.18	.029	
Extraversion	−.05	−5.84	<.001	
Openness	.02	1.77	.077	
DRS-15-R	.01	1.22	.224	

*Note.* Dependent variable: PHQ-ADS at T2. *DRS-15-R =* Revised Norwegian dispositional resilience scale, *PHQ-ADS =* Patient Health Questionnaire Anxiety and Depression Scale, *TIPI =* Ten-Item Personality-Inventory, *T =* Wave number. Results were essentially identical, with no differences in significance levels, when running the regression using multiple imputation (*N =* 5969; see supplemental Table 2). *p < .001

The Durbin-Watson statistic was 1.99 indicating nonappearance of autocorrelation. No issues with multicollinearity were found, as VIF values ranged from 1.03 to 1.91 Results were essentially identical, with no differences in significance levels, when the regression analysis was based on data imputed with multiple imputation (*N =* 5969; se supplemental Table 2).

### Hardiness as a moderator of the relationship between neuroticism and mental distress

Table 4 displays the results of the association between *neuroticism* and anxious-depressive symptomatology, moderated by *hardiness*. The overall model explained 61% of the variance in symptoms (*F* [13, 5769] = 746.06, *p <* .001, *R*^*2*^ *=* .61). The individual predictors and covariates *neuroticism*, age, solitary living, negative economic impact, mental distress at baseline, *conscientiousness*, *agreeableness*, and *extraversion* significantly predicted mental distress. The interactional effect between *neuroticism* and *hardiness* on mental distress was not significant. Addition of the interaction between *neuroticism* and *hardiness* did not significantly explain variance in mental distress (*F* [1, 5769] = .45, *p =* .500, Δ*R*^*2*^ *=* .000).

**Table 4:** Model coefficients in the moderation analysis (*N* = 5783)

Model coefficients	*b*	*SE*	*t*	*p*	*95% CI*
Neuroticism T2	1.62	.08	20.01	<.001	[1.457, 1.773]
Hardiness T1	.02	.02	1.13	.257	[−.017, .063]
Neuroticism x Hardiness	−.01	.01	−.67	.500	[−.024, .011]
Age T1	−.05	.01	−6.67	<.001	[−.058, −.031]
Female T1	.42	.23	1.85	.065	[−.026, .872]
Other gender T1	−1.12	1.44	−.78	.437	[−3.929, 1.698]
Solitary living T2	.54	.22	2.50	.012	[.117, 967]
Neg. econ. impact T2	1.80	.23	7.69	<.001	[1.338, 2.253]
PHQ-ADS T1	.55	.01	40.91	<.001	[.522, .575]
Conscientiousness T2	−.65	.09	−7.38	<.001	[−.818, −.475]
Agreeableness T2	−.20	.09	−2.12	.034	[−.376, −.015]
Extraversion T2	−.38	.07	−5.52	<.001	[−.511, −.243]
Openness T2	.15	.09	1.67	.095	[−.027, .332]

*Note*. Model fits are presented in text. *X* = Interaction, *T =* Wave number, *Neg. econ. impact* = Negative economic impact. Hardiness = Revised Norwegian dispositional resilience scale. Dependent variable = PHQ-ADS T2. Unstandardized *b-*coefficient

## Discussion

The present study set out to investigate if personality traits and *hardiness* could represent risk- and protective factors for mental distress during the COVID-19 pandemic. Specifically, the predictive validity of personality traits and *hardiness* for mental distress were investigated. A moderation analysis was also applied, with the intent to examine if *hardiness* buffered the effect of *neuroticism* on mental distress.

Regarding personality, all traits were significant and negatively correlated with mental distress at both T1 and T2, except for *neuroticism* which was significant and positively correlated with mental distress at both times of measurement. Personality traits at T2 uniquely explained 5% of the variance in symptoms at T2 when controlling for age, gender, solitary living, negative economic impact, and mental distress at baseline. *Neuroticism* constituted a risk factor for mental distress. Contrastingly, *conscientiousness, extraversion* and *agreeableness* were implicated as protective factors. O*penness* was not significantly associated with mental distress. Contrary to the study’s third hypothesis, the results did not find evidence for hardiness moderating the association between neuroticism and mental distress.

These results corroborate the existing literature of personality traits’ association to anxious-depressive symptomatology [11–13] in the context of the COVID-19 pandemic. As presented, *neuroticism* is most consistently related to such symptoms. Rumination and worry are described as cognitive manifestations of *neuroticism* [54, 55], and are implicated in the etiology of anxious and depressive disorders [56]. High *neuroticism* may result in an inclination towards worry and rumination, which in turn might contribute to presence of mental distress [54–56]. Alternatively, one might underscore the fact that the pandemic has involved a myriad of negative consequences and uncertainty on multiple levels, with impact on many people’s lives. *Intolerance of uncertainty* and *pessimistic inferential style* are known cognitive risk factors for anxiety and depression, respectively [57]. Hence, one might argue that individuals possessing one or both cognitive risk factors, in addition to a high score on *neuroticism*, might be especially vulnerable to worry and rumination, and thus anxious-depressive symptomatology, throughout uncertain and distressing times of the COVID-19 pandemic.

Pertaining to *hardiness*, the current results revealed that all three *hardiness* constructs were significant and negatively correlated with mental distress at both times of measurement. However, *hardiness* did not explain variance in outcome above and beyond personality traits. Furthermore, when treated as a single predictor in the last step of the regression, *hardiness* was not significant. This contradicts Eschleman and colleagues’ [40] results, which established significant, negative relationships between *hardiness* and depression and anxiety. Thus, the current findings accentuate the importance of controlling for personality traits when examining the predictive validity of *hardiness*.

As a theoretical construct, *hardiness* is postulated to protect against negative health-effects of stress. Thus, several studies have included some form of stress-measure [36–38] as an interactional variable. Consequently, the present study’s lack of a stress measure might explain why *hardiness* was insignificant in the regression- and moderation models. In congruence with this line of reasoning, Havnen et al. [27] found that resilience (using a different measure than the DRS-15-R) diminished the impact of stress on anxious-depressive symptoms during the COVID-19 pandemic. Other factors that might contribute to the contrasting findings of the current study and the study of Havnen et al. [27], might be different operationalizations of resilience, differences in sample sizes and sample characteristics, and/or that the current study controlled for personality traits.

This study’s final hypothesis was falsified, as hardiness did not significantly moderate the positive association between *neuroticism* and mental distress. The premises in the previous discussion of the lack of a stress measure in relation to *hardiness*, might also apply to explain the results of the moderation model. Consequently, we cannot rule out that *hardiness* may have significantly moderated the association between stress and mental distress during the pandemic. Moreover, very few subjects (*n =* 60) scored high (≥1 *SD* above *M*) on *neuroticism* and *hardiness* simultaneously. Similarly, only 39 participants scored low (≤1 *SD* below *M*) on both *hardiness* and *neuroticism* simultaneously. The negative correlation coefficients between *hardiness* and *neuroticism* (table 2) also indicates this tendency. This might aid explanation of the insignificant interactional effect in the moderation model, as individuals with high *neuroticism* seldomly had higher *hardiness* to buffer against symptoms.

Finally, the present study corroborates risk factors of anxious-depressive symptomatology in the existing COVID-19-literature (e.g., 2, 4, 5, 8), as the results demonstrated that young age, self-reported solitary living, and negative economic impact were significant predictors of mental distress. As highlighted by Brand [58], economic concerns are likely to cause mental distress. The current results validate this notion. Additionally, mental distress at T1 was the strongest predictor of mental distress at T2, corroborating the notion regarding stability in symptoms and the exposed situation of vulnerable groups during the COVID-19 pandemic. Moreover, this study corroborates the evidence implicating solitary living as a risk factor for mental distress during COVID-19 pandemic. Although the current results cannot explain why such living condition constitutes a risk factor, one might argue that for some solitary living contributes to unfulfilled social needs, less social support, and increased perceived social isolation during the COVID-19-related lockdown.

### Limitations

The current study has limitations that warrant consideration. First, utilizing convenience sampling limits the generalizability of the results. So does the low mean age, and overrepresentation of students and women in the sample. Second, the sampling procedures prohibits estimation of response rates, as the number of people invited to participate is unknown. Third, as brevity was a priority, the TIPI was applied to measure personality traits. However, the TIPI is psychometrically inferior to more comprehensive standard measures of personality traits. The internal consistency estimates of the TIPI were especially weak for two personality traits (openness and agreeableness) which could be expected when measuring traits using only two items. Fourth, individuals concerned with the pandemic might be more inclined towards participation. Hence, this study may be vulnerable to a certain degree of response bias. Fifth, attrition rates from T1 to T2 were high, which could inflate the risk of attritional bias. However, only minor differences in demographic variables and scores of mental distress were found between the two samples. Thus, the risk of attritional bias is minimal, although it cannot be completely ruled out. Sixth, although the TIPI and the DRS-15-R measures constructs on a dispositional level, one might argue that all predictor variables should have been measured at both times of measurement, resulting in a purely longitudinal study design. Seventh, there are some concerns regarding the discriminant validity between *hardiness* and personality traits, as both are dispositional variables. As accentuated by Oshio et al. [22], future studies should indeed resolve whether trait resilience (e.g., *hardiness*) should be regarded as a personality characteristic or not. Moreover, future research investigating the predictive capacity of *hardiness* should control for personality traits. Finally, the measures of alcohol consumption, exercise, job status, living conditions, psychiatric disorder, and economic impact were unstandardized, unvalidated self-report measures.

## Conclusion

This study has identified some potential predictors of mental distress 9 months after the outbreak of the COVID-19 pandemic in Norway. Symptoms at baseline, solitary living, perceived negative economic impact and *neuroticism* emerged as potential risk factors for mental distress. *Conscientiousness* and *extraversion* were the most pronounced protective factors for mental distress.

The current findings could aid identification of vulnerable groups and individuals (i.e., groups and individuals who possess one or more of the identified risk factors, and few or none of the identified protective factors) throughout the pandemic. These groups/individuals might profit from various preventive measures, at multiple levels of intervention. Such preventive interventions might include (non-exhaustive list) universal, selective and/or indicative psychosocial, educational, occupational, recreational and/or economic measures. Interventions could be delivered at individual-, group-, and/or population-level. Individuals with abnormal levels of anxious-depressive symptomatology, should receive the appropriate, evidence-based biopsychosocial treatment. The National Institute for Health and Care Excellence (NICE) recommends cognitive behavioral therapy in the treatment of depression and anxiety [59, 60].

## Supplementary Information

**Additional file 1.**

## Data Availability

The dataset used and analyzed during the current study is available from the corresponding author on reasonable request.

## Abbreviations

BDI: Beck Depression Inventory
COVID-19 pandemic: The pandemic caused by the virus entitled Severe acute respiratory syndrome coronavirus type 2
DRS-15: Dispositional Resilience scale-15
DRS-15-R: Revised Norwegian Dispositional Resilience Scale
GAD-7: Generalized Anxiety Disorder 7
PHQ-9: Patient Health Questionnaire-9
PHQ-ADS: Patient Health Questionnaire Anxiety and Depression Scale
T1: First time of measurement
T2: Second time of measurement
TIPI: Ten-Item Personality-Inventory
VIF: Variance inflation factor
