# Supplementary material for: Personality traits and hardiness as risk- and protective factors for mental distress during the COVID-19 pandemic: a Norwegian two-wave study
Source: BMC Psychiatry. 2022 Sep 15;22:610. doi: 10.1186/s12888-022-04237-y (PMC9476397; doi:10.1186/s12888-022-04237-y)
Supplement: Supplementary file 1 — Additional file 1. [file 12888_2022_4237_MOESM1_ESM.docx]

**Supplemental table 1**

*Correlations between hardiness, personality traits, and mental distress (N = 5,969)*

| Variable | 1 | 2 | 3 | 4 | 5 | 6 | 7 | 8 | 9 | 10 |
| --- | --- | --- | --- | --- | --- | --- | --- | --- | --- | --- |
| 1. Commitment T1 |  |  |  |  |  |  |  |  |  |  |
| 2. Control T1 | .37^*^ |  |  |  |  |  |  |  |  |  |
| 3. Challenge T1 | .33^*^ | .16^*^ |  |  |  |  |  |  |  |  |
| 4. DRS total T1 | .81^*^ | .66^*^ | .70^*^ |  |  |  |  |  |  |  |
| 5. Neuroticism T2 | -.38^*^ | -.21^*^ | -.35^*^ | -.44^*^ |  |  |  |  |  |  |
| 6. Extraversion T2 | .34^*^ | .16^*^ | .32^*^ | .39^*^ | -.21^*^ |  |  |  |  |  |
| 7. Agreeableness T2 | .17^*^ | .09^*^ | .13^*^ | .19^*^ | -.20^*^ | .13^*^ |  |  |  |  |
| 8. Openness T2 | .24^*^ | .11^*^ | .34^*^ | .32^*^ | -.15^*^ | .31^*^ | .18^*^ |  |  |  |
| 9. Conscientiousness T2 | .32^*^ | .17^*^ | .08^*^ | .27^*^ | -.26^*^ | .16^*^ | .21^*^ | .07^*^ |  |  |
| 10. PHQ-ADS T1 | -.57^*^ | -.26^*^ | -.37^*^ | -.56^*^ | .54^*^ | -.21^*^ | -.12^*^ | -.10^*^ | -.27^*^ |  |
| 11. PHQ-ADS T2 | -.47^*^ | -.20^*^ | -.30^*^ | -.46^*^ | .58^*^ | -.23^*^ | -.16^*^ | -.10^*^ | -.31^*^ | .74^*^ |

*Notes.* Based on multiple imputation. Variables 1-4 = revised Norwegian dispositional resilience scale; variables 5-9 = Ten-Item Personality-Inventory; PHQ-ADS = Patient Health Questionnaire Anxiety and Depression Scale; T = wave number. Of the 6017 subjects participating in T2, 48 were trimmed before analyses, due to an unreliable reporting style. This number diverges from the 41 participants trimmed due to an inconsistent reporting style in the main analyses. This difference is caused by an overlap of 7 participants with missing values on the anxiety- and depression measures, as well as an unreliable response style. Hence, the 7 participants were trimmed as a part of the PHQ-ADS-trimming procedures, and thus not in the inconsistent reporting-trimming procedures, in the main analyses.

**p <* .001**Supplemental table 2**

*Predicting symptom of anxiety and depression (T2) using personality traits and hardiness (N = 5,969)*

| Step | *F* | *p* | *R^2^_adj._* | *ΔR^2^* |
| --- | --- | --- | --- | --- |
| 1. Age & gender T1 | 232.21 | <.001 | .10 | .11* |
| 2. Solitary living T2 | 192.81 | <.001 | .11 | .01* |
| 3. Negative economic impact T2 | 218.74 | <.001 | .15 | .04* |
| 4. PHQ-ADS T1 | 1277.33 | <.001 | .56 | .41* |
| 5. TIPI T2 | 857.37 | <.001 | .61 | .05* |
| 6. DRS-15-R T1 | 786.18 | <.001 | .61 | .00 |
|  |  |  |  |  |
| Predictors in final step | *B* | *SE* | *t* | *p* |
| Age | -.05 | .01 | -6.93 | <.001 |
| Female | .34 | .23 | 1.49 | .135 |
| Other gender | -1.84 | 1.20 | -1.54 | .125 |
| Solitary Living | .48 | .21 | 2.27 | .023 |
| Negative economic impact | 1.79 | .21 | 8.50 | <.001 |
| PHQ-ADS | .54 | .01 | 47.81 | <.001 |
| Neuroticism | 1.65 | .07 | 22.38 | <.001 |
| Conscientiousness | -.67 | .08 | -8.60 | <.001 |
| Agreeableness | -.19 | .09 | -2.16 | .031 |
| Extraversion | -.37 | .06 | -5.75 | <.001 |
| Openness | .16 | .09 | 1.93 | .053 |
| DRS-15 | .02 | .02 | 1.10 | .271 |

*Note.* Dependent variable: PHQ-ADS at T2. DRS-15-R = revised Norwegian dispositional resilience scale; PHQ-ADS = Patient Health Questionnaire Anxiety and Depression Scale; TIPI = Ten-Item Personality-Inventory, T = wave number. Female = female (coded 1) vs. male and other (coded 0); Other gender = other (coded 1) vs. male and female (coded 0). Based on multiple imputation. Durbin-Watson = 1.99. VIF = 1.03-1.92.

**p <* .001
